# MartiniSurf: Automated Simulations of Surface-Immobilized Biomolecular Systems with Martini

**DOI:** 10.1021/acs.jcim.6c00953

**Published:** 2026-06-16

**Authors:** Juan Carlos Jiménez-García, Fernando López-Gallego, Xabier López, David De Sancho

**Affiliations:** † Polimero eta Material Aurreratuak: Fisika, Kimika eta Teknologia, Kimika Fakultatea, 226245 UPV/EHU & Donostia International Physics Center (DIPC), PK 1072, 20018 Donostia-San Sebastián, Euskadi, Spain; ‡ Center for Cooperative Research in Biomaterials (CIC biomaGUNE), 90216 Basque Research and Technology Alliance (BRTA), Paseo Miramon 194, 20014 San Sebastián, Spain; § Polimero eta Material Aurreratuak: Fisika, Kimika eta Teknologia, Kimika Fakultatea, UPV/EHU and Donostia International Physics Center (DIPC), PK 1072, 20018 Donostia-San Sebastian, Euskadi, Spain

## Abstract

The rational design of biomolecule immobilization strategies requires molecular-level understanding of how surface properties, tethering geometry, and structural dynamics jointly influence stability and function. Recently, coarse-grained molecular dynamics simulations based on the Martini force field have emerged as an efficient framework for studying enzyme–surface interactions. However, the reproducible construction of immobilized systems with controlled orientations remains technically challenging, usually involving multiple computational tools. Here we present MartiniSurf, an open-source command-line package for the preparation of protein and DNA systems immobilized on solid supports within the Martini paradigm. MartiniSurf integrates automated structure retrieval and cleaning, coarse graining via tools from the Martini force field software ecosystem, customizable surface generation, and biomolecule orientation based on user-defined anchoring residues, producing complete GROMACS-ready simulation systems. The package supports both implicit restraint-based anchoring and explicit linker-mediated immobilization, including surfaces functionalized with user-defined ligands or linker-like moieties, enabling representation of mono- and multivalent attachment geometries at different modeling resolutions. Structure-based Go̅Martini potentials can be incorporated for proteins, while DNA systems are modeled using Martini 2. Optional substrate insertion, pre-coarse-grained complex handling, and automated solvation and ionization further extend system flexibility. By integrating these components into a unified workflow, MartiniSurf enables reproducible preparation and *in silico* exploration of surface-tethered biomolecular systems.

## Graphical abstract

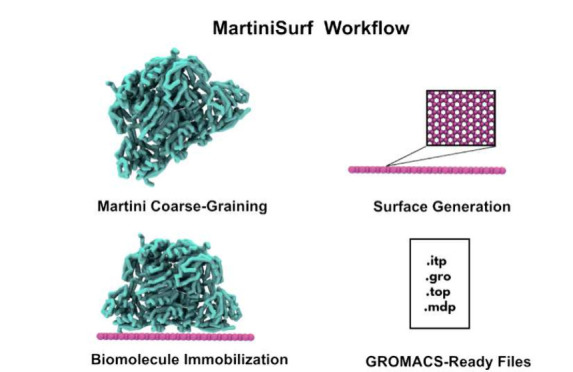

## Introduction

Despite the widespread use of biomolecule immobilization in biotechnology, developing systematic strategies to identify surface tethering sites remains a challenge. Even when identical surface chemistries are used, variations in the attachment site, orientation, or tethering geometry can lead to markedly different functional outcomes.^1–4^ As a result, there is growing interest in site-directed rational immobilization approaches that aim to control molecular orientation and local rigidity while preserving access to active regions.^5–7^ Molecular dynamics (MD) simulations offer a powerful complementary approach for probing biomolecule–surface interactions at molecular resolution. All-atom simulations, in particular, have yielded detailed insights into adsorption mechanisms, orientation preferences, and surface-induced conformational changes.^8–10^ However, their high computational cost limits systematic exploration across multiple immobilization geometries, surface models, and biomolecular variants, particularly when long time scales or large assemblies are required. Coarse-grained (CG) models address this limitation by reducing molecular resolution while retaining essential physicochemical features.^11^ Among these, the Martini force field has emerged as a widely adopted framework for large biomolecular systems.^12–16^ When combined with structure-based Go̅ like potentials, Martini simulations can preserve native folds while enabling collective motions relevant to biomolecular function.^17^ Recently, we have shown that structure-based Martini simulations can provide qualitative mechanistic insight into enzyme immobilization, including the coupling between tethering geometry, conformational dynamics, and catalytic function.^18^ In that study, alcohol dehydrogenase (ADH) immobilized on an agarose-like surface was simulated using the Go̅Martini model.^17^ Variants involving different immobilization sites and strategies were modeled through multivalent harmonic restraints on an idealized agarose-like surface, revealing orientation-dependent modulation of flexibility and substrate accessibility. However, the system construction required the multiple configuration steps involving manual preprocessing, custom scripts, and nonstandardized orientation procedures. In particular, enforcing reproducible, orientation-controlled, and multivalent anchoring strategies — which are central to modern experimental immobilization approaches^6,19^ — remains technically challenging. This highlights the need for a unified and reproducible framework capable of systematically generating such immobilized simulation systems and extending them toward more complex surface chemistries.

To address these limitations, here we present MartiniSurf, an automated framework that consolidates the reproducible construction of surface-immobilized biomolecular systems within the Martini paradigm, enabling systematic *in silico* exploration of immobilization geometries at coarse-grained resolution (Figure 1).

**Figure 1:**
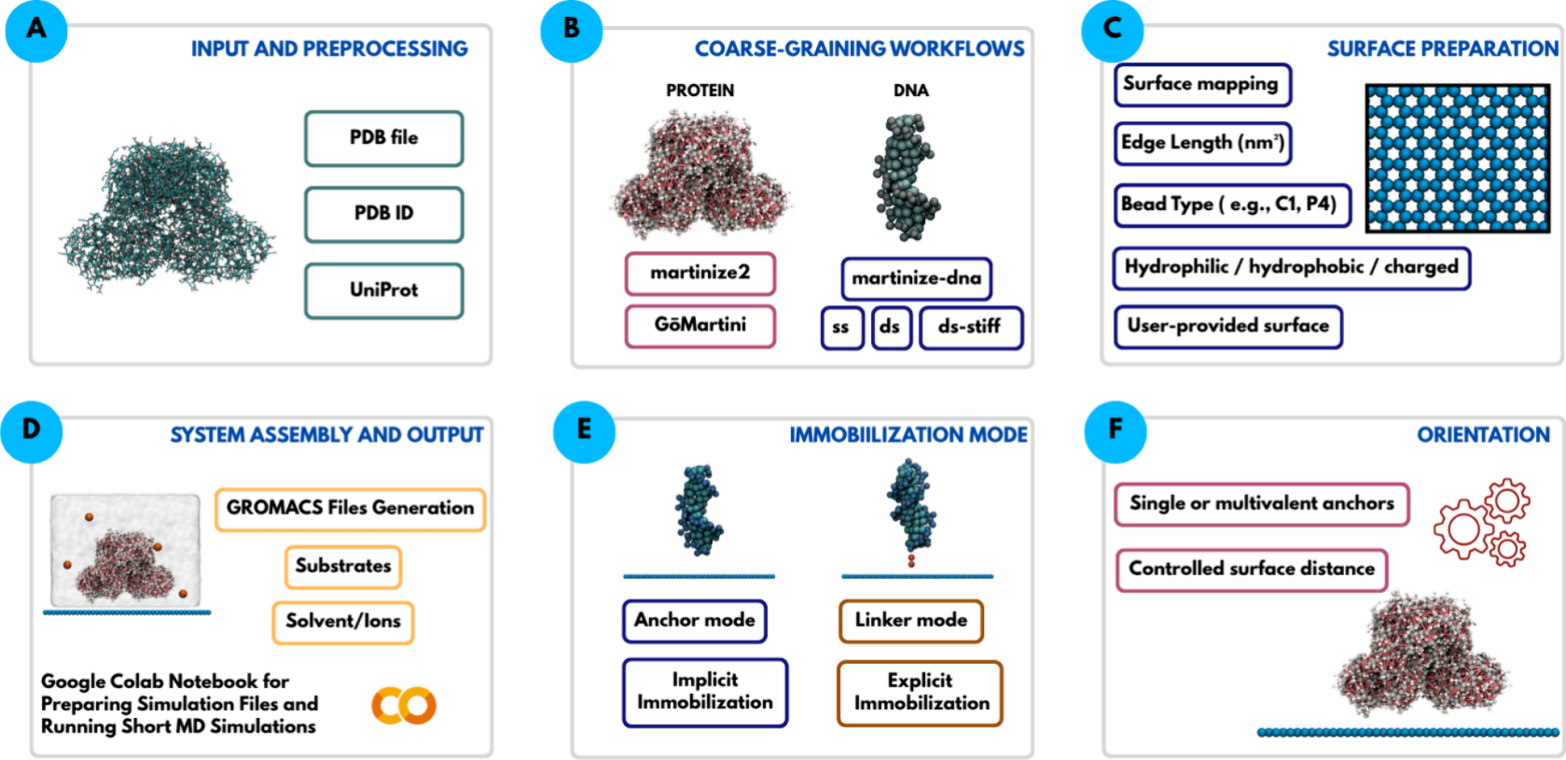
Overview of the MartiniSurf workflow for constructing surface-immobilized biomolecular systems for coarse-grained molecular dynamics. (A) Biomolecular input structures can be provided as local coordinate files, RCSB PDB identifiers, or UniProt accessions. (B) MartiniSurf performs structure preprocessing and applies dedicated coarse-graining workflows for proteins or DNA. (C) Model surfaces are generated with tunable lattice geometry, bead chemistry, and optional functionalization. (D) The biomolecule is oriented relative to the interface using anchor- or linker-based immobilization strategies. (E) The oriented biomolecule, surface, optional linkers, solvent, ions, and substrates are assembled into a complete simulation system. (F) The workflow produces GROMACS-ready files and associated Colab resources for short molecular-dynamics simulations and visual inspection.

## Methods and Implementation

MartiniSurf was designed around four core requirements relevant to computational studies of biomolecule immobilization. First, *automation*: the complete system-construction process should be executable from the command-line interface (ideally using a single command), minimizing manual intervention and user-dependent variability. Second, *reproducibility*: explicit input parameters define the generated system, enabling robust comparison across immobilization geometries and independent studies. Third, *flexibility*: the framework should support different types of biomolecule, force-field variants, surface models, and tethering strategies without requiring custom code modifications. Fourth, *extensibility*: the framework should be readily adaptable to incorporate new functionalities and evolving Martini model developments, ensuring long-term usability. Together, these design principles enable MartiniSurf to prepare complete simulation systems for downstream simulations using the popular molecular mechanics package GROMACS.^20^

### Coarse Graining with Tools from the Martini Ecosystem

The workflow begins from a user-provided biomolecular structure specified through the --pdb flag, which accepts local .pdb, .cif, or .mmcif coordinate files, a 4-character RCSB PDB identifier, or a 6-character UniProt accession used to retrieve the corresponding AlphaFold structural model. Input structures are then cleaned and standardized prior to coarse graining.

Protein systems are processed using martinize2,^21^ whereas DNA systems are handled using the established Martini 2 martinize-dna.py program.^22^ At the command-line level, MartiniSurf provides the main options required to select and configure these workflows, while preserving a unified setup interface for proteins, nucleic acids, and pre-coarse-grained assemblies.

For protein systems, MartiniSurf uses Martini 3 through martinize2; in this work, the tested implementation was that of the martinize2 program available in the Vermouth package (version 0.15.0) using the Martini 3 force-field option -ff martini3001. The main protein setup options are provided through flags such as --moltype, --merge, --p, --pf, --dssp, --elastic, --ef, and --go. These options control molecule naming, chain merging, position restraints, DSSP-based secondary-structure assignment,^23^ elastic-network stabilization, and structure-based Go̅Martini biasing. In particular, --elastic enables elastic-network stabilization, whereas --go activates the Martini 3-compatible virtual-site-based Go̅Martini model implemented in martinize2.^15^ When no external Go̅ contact map is provided, MartiniSurf relies on the default martinize2 contact-map generation procedure, which identifies native contacts using residue-overlap (OV) and restricted chemical structural-unit (rCSU) criteria, followed by filtering based on backbone-bead distance and residue graph separation.^15,17^ Additional Go̅Martini-related options, such as --go-eps, --go-low, --go-up, and --go-res-dist, allow users to control the bias strength and contact-filtering criteria propagated from the underlying martinize2.

For DNA systems, the --dna flag activates the Martini 2 DNA workflow, while --dnatype selects the nucleic-acid topology class used by martinize-dna.py. This workflow is separate from the protein martinize2 pathway and currently requires Python 2.7, reflecting the requirements of the underlying DNA coarse-graining script. MartiniSurf currently exposes several DNA setup modes, including ds-stiff, ds-soft, ss, and ss-stiff. The ds-stiff mode, used as the default for double-stranded DNA, relies on the elnedyn22dna force field with a stiff elastic-network setup, whereas ds-soft uses a softer elastic network. The ss mode uses the martini22dna force field for single-stranded DNA, while ss-stiff extends single-stranded DNA setup with additional stiffness through the underlying elastic-network treatment. Additional modes supported by the underlying script, such as all-soft and all-stiff, can also be used for multichain systems.

Pre-coarse-grained biomolecular assemblies can be incorporated through the --complex-config option, which bypasses the martinization stage and directly enters the surface-orientation and assembly workflow. This is useful for systems that already contain externally generated Martini-compatible topologies, cofactors, ligands, linkers, or multicomponent biomolecular complexes.

Thus, MartiniSurf provides a unified high-level interface for atomistic protein inputs, DNA structures, and prebuilt coarse-grained assemblies. The generation of biomolecular coarse-grained topologies, elastic-network definitions, Go̅Martini contact maps, and DNA model parameters remains governed by the corresponding Martini ecosystem tools or by externally supplied files. MartiniSurf propagates the selected user options, preserves the generated components, and assembles them into complete GROMACS-ready surface-immobilized systems.

### Surface Generation

Following coarse graining, MartiniSurf generates model solid supports represented as Martini-bead surfaces, including built-in planar reference supports based on two-dimensional hexagonal lattices. In practice, the built-in planar surface types are selected through the --surface-mode flag, including options such as 4–1 (honeycomb planar lattice) or 2–1 (hexagonal planar lattice). The lattice spacing, bead identity, charge distribution, number of layers, stacking sequence, surface-density mode, and optional surface functionalization can be explicitly defined. For multilayer local 2–1 and 4–1 surfaces, MartiniSurf uses an HCP-like ABAB stacking by default, while the --surface-stacking fcc option generates FCC-like ABCABC stacked lattices. For generic lattice-based supports, the surface bead identity can be selected by the user through --surface-bead; in Martini 3 this includes standard bead classes such as C1–C6, N1–N6, and P1–P6. In particular, MartiniSurf supports the addition of user-defined ligands or linker-like moieties to the surface through the --surface-linkers option, enabling the construction of functionalized coarse-grained interfaces beyond bare reference supports.

Earlier Martini 2-based studies explored lattice-based coarse-grained surfaces to model carbon-based and chemically tuned materials. In these approaches, bead-type adjustments were used to capture the effects of surface chemistry,^24^ while alternative mapping schemes, such as 4:1 and 2:1 representations, were proposed for graphitic materials to capture surface density and interaction properties.^25,26^ Extensions to chemically heterogeneous graphene oxide surfaces combining modified bead types and reduced mapping schemes were also reported.^27^ Similar hexagonal lattice strategies were used to represent polysaccharide-based materials, such as agarose-like surfaces.^28^ MartiniSurf incorporates these lattice-based strategies into a reproducible setup workflow, allowing users to systematically vary surface-related parameters within the limits of the selected Martini parametrization.

Recent Martini 3-based studies of solid–liquid interfaces have emphasized that surface model construction requires particular care.^29,30^ Cambiaso et al. showed that highly regular Martini 3 solid surfaces based on compact FCC (111) lattices, can promote excessive ordering of interfacial water, leading to artificial water layering and surface-induced freezing-like artifacts.^30^ They further showed that these artifacts can be mitigated or tuned by introducing static or dynamic surface disorder, adjusting the lattice spacing and surface bead density, and using mixtures of Martini 3 water beads of different sizes (W, SW, and TW), with higher fractions of tiny water beads helping to reduce interfacial ordering. In the same work, this lattice-based strategy was then used to construct Martini 3 silica surface models by selecting suitable N-type beads and tuning the lattice spacing/surface density to reproduce target interfacial properties such as water contact angles. These considerations can be explored in MartiniSurf through tunable bead type, lattice spacing, surface-density mode, stacking, surface disorder, and solvent composition.

MartiniSurf allows the final Martini 3 solvent composition to be tuned through the --water-mix option, which converts user-defined fractions of standard W beads into SW and/or TW beads after solvation. For example, --water-mix SW:0.10,TW:0.10 generates a final solvent composition containing 80% W, 10% SW, and 10% TW. This provides a simple route to explore water-bead-size mixtures in Martini 3 surface systems, while leaving the underlying Martini water parameters unchanged.

Within the built-in surface-generation workflow, graphene and graphite are available through the corresponding --surface-mode options, whereas carbon nanotubes are generated through dedicated CNT modes (cnt-m2 and cnt-m3) in the surface-building module.^31^ The force-field version used for these supports is selected consistently with the biomolecular workflow: Martini 3-compatible graphene and graphite models are used for protein systems,^32^ whereas Martini 2-compatible graphene and graphite models are used for DNA systems.^25,26^ Carbon nanotubes can be generated using either Martini 2 or Martini 3 representations depending on the selected workflow, while fullerene models are currently implemented only for Martini 3.^32^ Rather than introducing new parametrizations, MartiniSurf reuses and adapts previously reported coarse-grained models and code for carbon nanomaterials,^31,32^ enabling their use in biomolecule-immobilization workflows.

Likewise, saccharide-rich supports such as agarose can be represented using dedicated Martini 3 carbohydrate parametrizations,^33^ allowing more detailed coarse-grained representations of polysaccharide-based materials beyond idealized lattice models. In this way, MartiniSurf combines idealized reference surfaces with previously parametrized carbon-based material models while remaining compatible with emerging Martini-based material representations.

### Biomolecule Orientation

Beyond combining existing Martini coarse-graining tools and surface models, a central component of MartiniSurf is its orientation engine, which aligns user-defined tethering residues toward the surface at a prescribed separation distance through rigid-body transformations. This functionality is implemented through three CLI-defined surface-coupling regimes: anchor-based immobilization, in which selected residues (--anchor) are restrained at a distance (--dist) relative to the surface; explicit linker-based immobilization (--linker, --linker-group), in which attachment is mediated by a coarse-grained linker; and adsorption mode (--ads-mode), which orients the biomolecule toward the surface without imposing an explicit anchoring setup. For example, multivalent protein immobilization can be defined by providing multiple --anchor selections, where the first argument is the chain identifier and the following arguments are residue numbers, e.g., --anchor A 8 10 11 --anchor D 8 10 11 anchors residues 8, 10, and 11 from chains A and D. DNA tethering can instead be specified through an explicit aliphatic linker using --linker together with a residue selection, for example --linker-group A 1, which assigns the linker to residue 1 of chain A. Because these surface-coupling modes are fully parameter-driven and defined before solvation and ion addition, identical input configurations generate reproducible immobilization geometries while allowing precise control over mono- or multivalent attachment schemes.

Within this framework, anchor and linker modes provide complementary representations of immobilization. In *anchor mode*, surface attachment is represented through harmonic potentials applied between user-defined anchoring residues and the support. This mode originates from the harmonic-tethering protocol developed and applied in our previous study of oriented alcohol dehydrogenase immobilization, where selected anchoring residues were restrained to an agarose-like surface to model experimentally motivated immobilization geometries.^18^ In *linker mode*, an explicit tether molecule is introduced between the surface and the biomolecule, allowing linker geometry, flexibility, and surface functionalization to be modeled directly, and enabling systematic exploration of how linker architecture and surface chemistry influence protein–surface coupling. Linker properties have been shown to play a critical role in modulating enzyme behavior at solid interfaces; for example, enzyme thermodynamics can be strongly affected by linker length and flexibility.^34^

Because linker molecules and surface-bound functional groups are treated as explicit coarse-grained components, their use requires prior Martini-compatible parametrization, including appropriate bead types, bonded terms, and interaction parameters consistent with the target force-field variant. In this context, automated small-molecule coarse-graining tools such as AutoMartini for Martini 2 ^35^ and recent Martini 3 workflows^36^ provide a practical route for generating linker topologies compatible with the Martini framework and can facilitate the incorporation of chemically resolved surface modifications into MartiniSurf workflows.

Representative systems generated with our package are shown in Figure 2. Panel (A) illustrates immobilization of the BsADH-H3 variant on an agarose-like surface via multivalent harmonic restraints (residues H8, H10, and H11), corresponding to the configuration analyzed previously^18^ Panel (B) extends this setup toward explicitly functionalized surfaces, where surface properties become adjustable structural parameters. Panel (C) presents the complete catalytic assembly constructed with MartiniSurf, including the enzyme, the NADH cofactor,^37^ and inserted ethanol substrate molecules, mirroring the enzyme-cofactor-substrate system examined in our prior work.^18^ These examples illustrate how the same orientation and assembly rationale can be applied to restrained, functionalized, and substrate-containing immobilized enzyme systems.

**Figure 2:**
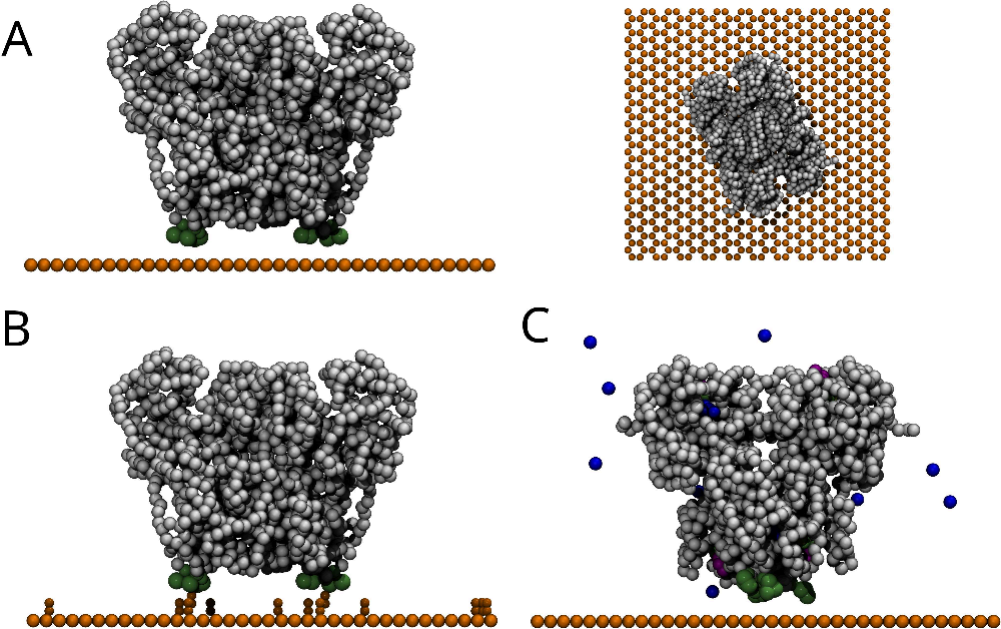
Surface-immobilized ADH systems built with MartiniSurf. (A) Immobilization on an agarose surface (side and top view). (B) Chemically functionalized surface with explicit surface-bound groups. (C) Complete catalytic system including NADH and inserted ethanol molecules. The enzyme is shown in white, the surface beads in orange, anchoring residues in green, surface functional groups in yellow, NADH in purple, and ethanol molecules in blue.

### Extension to DNA Immobilization

Surface-immobilized nucleic acids are also of broad interest in biotechnology, particularly in applications such as biosensing, nanobiotechnology, and DNA-based catalysis.^38,39^ In this context, aptamers — short DNA or RNA oligonucleotides capable of specifically binding molecular targets — represent an important class of surface-functional biomolecules, as their performance often depends on how tethering and surface proximity affect structure and accessibility. This makes nucleic-acid immobilization a relevant test case for extending MartiniSurf beyond enzyme–surface systems.

To assess the applicability of MartiniSurf to surface-immobilized nucleic-acid systems, we constructed a coarse-grained model reproducing the DNA–graphene setup reported by Kabeláč et al.^40^ In this workflow, the Martini 2 DNA setup was combined with explicit tethering through --linker and --linker-group. The decamer sequence 5’-CCACTAGTGG-3’ was modeled using the Martini 2 DNA representation, explicitly including at the coarse-grained level the C6 aliphatic linker (−(CH_2_)_6_−) attached to the 5’ terminus. In the DNA–surface workflow, MartiniSurf includes harmonic bonded terms between neighboring surface beads by default. This maintains the surface lattice while allowing limited local fluctuations, following the rationale that dynamic surface disorder can help mitigate excessive water ordering at Martini solid–liquid interfaces.^30^

In Figure 3A, we show the two systems evaluated here, namely DNA immobilized on uncharged graphene and on positively charged graphene. The positively charged surface does not correspond to a specific experimental material, but rather follows the model introduced by Kabeláč et al.^40^ to probe the effect of surface charge on the orientation of a tethered oligonucleotide. Here, it is used as a controlled perturbation to test the sensitivity of the coarse-grained setup to changes in surface electrostatics.

**Figure 3:**
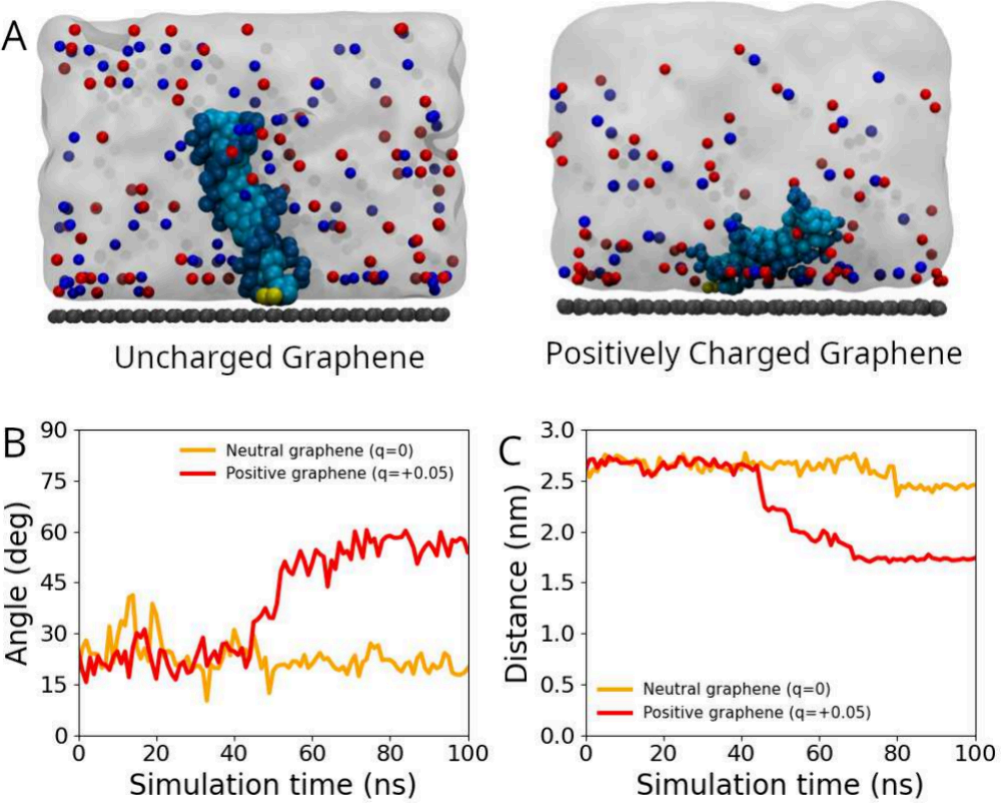
Structural stability and orientation of the surface-immobilized DNA. (A) Representative snapshot of the immobilized DNA on a graphene-like surface. The DNA is shown in a blue-cyan gradient, the surface beads in gray, and the linker in yellow. (B) Time evolution of the tilt angle between the DNA axis and the surface normal (*Z*-axis), where 0° corresponds to a perpendicular orientation and 90° to a parallel configuration. (C) Distance between the DNA center of mass and the graphene surface.

The coarse-grained simulations reproduce the main trends reported in the all-atom study at a qualitative level.^40^ In the neutral graphene system, the DNA remains predominantly oriented away from the surface, maintaining a relatively small tilt angle and a stable center-of-mass distance. In contrast, on positively charged graphene, the DNA progressively bends toward the surface, showing an increase in tilt angle and a reduction in the DNA–surface distance (Figure 3B,C). These observables provide a simple quantitative characterization of the orientational response, while the comparison is limited to reproducing the direction of the change rather than matching absolute values.

Overall, these results demonstrate that MartiniSurf-generated systems capture the expected charge-dependent orientational behavior of tethered DNA, consistent with previous atomistic simulations. This example is therefore intended as a controlled validation of the workflow and of the sensitivity of Martini 2 DNA models to surface properties, rather than as a quantitative validation of DNA–graphene interactions or as a source of new mechanistic insight.

### Command-Line Syntax and Output

MartiniSurf is invoked through a unified command-line interface in which the user specifies the biomolecular input, surface-construction parameters, orientation mode, and optional postprocessing steps through dedicated flags. For clarity, the main MartiniSurf command-line flags can be grouped according to their functional role in the setup workflow, as summarized in Table 1.

**Table 1:** Representative MartiniSurf Flags by Function

function	main flags
input structure	--pdb, --complex-config
protein setup	--moltype, --go, --go-eps, --go-low, --go-up, --go-res-dist, --elastic, --ef, --dssp, --martinize-extra-args
DNA setup	--dna, --dnatype, --merge, --p, --pf, --elastic, --ef
surface definition and functionalization	--surface-mode, --surface-bead, --dx, --lx, --ly, --surface-layers, --surface-stacking, --surface-linkers, --water-mix
anchor-based immobilization	--anchor, --dist
linker-based immobilization	--linker, --linker-group
adsorption setup	--ads-mode
system completion	--solvate, --ionize, --salt-conc
DNA solvent setup	--freeze-water-fraction, --freeze-water-seed

The final output of MartiniSurf is a fully assembled, GROMACS-ready simulation directory containing coordinate files, topology definitions, index groups, anchoring or linker restraint files when applicable, and template molecular dynamics parameter (MDP) files. The default output structure is organized into topology, MDP, and system folders, allowing immediate integration into downstream equilibration and production workflows.

### Representative Command-Line Examples

Before presenting representative commands, Table 2 summarizes the minimal inputs and main outputs for the two example workflows discussed below.

**Table 2:** Minimal Inputs and Main Outputs for Representative MartiniSurf Workflows

workflow	minimal inputs	main outputs
protein anchor-based immobilization	protein structure; selected anchoring residues; surface mode, bead type, and dimensions; solvation/ionization options.	oriented protein–surface system; Martini protein topology from martinize2; anchor restraints; GROMACS coordinate, topology, index, and MDP files
DNA linker-based immobilization	DNA structure; DNA topology type; linker coordinate/topology files; surface mode, bead type, and dimensions; solvation/ionization options.	oriented DNA–linker–surface system; Martini 2 DNA topology from martinize-dna.py; assembled GROMACS files; optional frozen-water setup

Representative MartiniSurf workflows can be specified using a small number of high-level flags. For example, anchor-based protein immobilization with solvation and ionization can be generated with the following command: martinisurf --pdb inputs/1RJW.pdb --dssp --go --moltype Protein --surface-mode 4–1 --surface-bead P4 --dx 0.47 --lx 15 --ly 15 --anchor A 8 10 11 --anchor D 8 10 11 --dist 1.0 --solvate --ionize --salt-conc 0.15 --merge A,B,C,D

A DNA linker-based workflow including solvation, ionization, and frozen water can be generated using with the following command: martinisurf --dna --dnatype ds-stiff --pdb inputs/4C64.pdb --surface-mode 4–1 --lx 10 --ly 10 --dx 0.27 --surface-layers 2 --surface-bead C1 C1 --linker inputs/ALK.gro --linker-group A 1 --solvate --ionize --salt-conc 0.15 --freeze-water-fraction 0.10 --freeze-water-seed 42 --merge A,B

## Applications and Outlook

MartiniSurf enables the systematic construction of surface-immobilized Martini systems by combining established biomolecular coarse-graining tools and surface models with automated orientation, tethering, and assembly procedures. By lowering the technical barrier to preparing these systems, the framework allows users to vary anchoring geometry, surface lattice properties, bead chemistry, and linker architecture in a reproducible manner. This makes MartiniSurf useful for exploring how immobilization setup choices may affect conformational stability, dynamic coupling, and catalytic accessibility in surface-bound enzymes.

The current implementation supports both idealized planar reference surfaces and previously parametrized carbon-based materials, including graphene, graphite, and carbon nanotubes in Martini 2- or Martini 3-compatible representations. This combination enables controlled comparisons on simplified supports as well as system preparation on chemically relevant graphitic materials within the same workflow. In addition, MartiniSurf can accommodate externally parametrized surfaces and linker-like components, making it adaptable to alternative surface architectures or heterogeneous materials when suitable Martini-compatible parameters are available.

Future developments may incorporate additional surface chemistries, dynamically flexible supports, optimized contact-map definitions for Go̅Martini models,^41^ alternative structure-preserving schemes such as OLIVES for Martini proteins,^42^ and automated postprocessing and analysis pipelines. In principle, similar extensions could also be considered for softer lipid-based interfaces, given the broad use of Martini in lipid bilayer simulations.^12,13,43^

In its current form, MartiniSurf is demonstrated for protein and DNA immobilization on Martini bead-based supports, including agarose-like and carbon-based surfaces, using anchor-based and linker-mediated coupling strategies. Future extensions will focus on more complex surface architectures, additional surface chemistries, and expanded automated analysis workflows.

## Implementation

MartiniSurf is implemented in Python and distributed as an open-source package. The codebase follows a modular architecture in which structure processing and coarse graining, surface construction, orientation, and final system assembly are handled by separate components coordinated through a high-level command-line interface. This separation improves maintainability, facilitates extension to new surface geometries and tethering strategies, and supports automated or high-throughput simulation workflows.

The workflow interfaces with established external programs, including martinize2 for proteins,^21^ martinize-dna.py for nucleic acids,^22^ DSSP,^23^ MDTraj,^44^ and GROMACS.^20^ Accordingly, MartiniSurf acts as a system-construction layer that combines the outputs of these tools with surface, linker, solvent, ion, and restraint components, rather than reimplementing biomolecular coarse graining, bias-model generation, or molecular-dynamics engines.

In this architecture, external Martini tools define the biomolecular coarse-grained models, whereas MartiniSurf provides the system-building logic required to construct, orient, tether, functionalize, and assemble surface-immobilized configurations in a reproducible manner.

### Reproducibility and Scope

MartiniSurf focuses on reproducible system construction and assembly, rather than force-field parametrization. To improve reproducibility, each generated simulation directory includes a provenance.json file recording the command-line arguments, workflow mode, relevant input paths, selected model parameters, detectable software versions, and executable paths. This includes the Python environment, key Python packages, and external programs such as martinize2, GROMACS, Python 2.7, and DSSP/mkdssp when available. The file also records whether the system was prepared through the protein, DNA, or pre-coarse-grained complex workflow, together with the selected surface, orientation, solvation, ionization, elastic-network, and Go̅Martini options.

In the representative software environment used to test the workflows reported here, MartiniSurf was run with Python 3, martinize2 program available in the Vermouth package (version 0.15.0), MDTraj 1.10.3, MDAnalysis 2.7.0, NumPy 1.26.4, SciPy 1.17.1, PyVista 0.46.4, and GROMACS 2020.1. The DNA workflow additionally requires Python 2.7 for the martinize-dna.py script. These versions define the tested software context rather than strict compatibility limits.

In terms of scope, MartiniSurf does not automatically parametrize arbitrary new chemical species. User-provided linkers, substrates, cofactors, or custom surfaces must be supplied with Martini-compatible topology files and parameters appropriate for the selected force-field version. MartiniSurf can place, orient, merge, and assemble these components into the final GROMACS topology, but the physical validity of externally supplied parametrizations remains the responsibility of the user.

### Colab Notebooks

To improve accessibility and interoperability within the Martini ecosystem, MartiniSurf also provides cloud-based Google Colab notebooks for protein and DNA system preparation. These interactive environments include auxiliary automated workflows for Martini 2 ^35^ and Martini 3 ^36^ small-molecule coarse graining, enabling atomistic-to-coarse-grained mapping, bead-type assignment, and topology generation without requiring local software installation. In addition to system building, the notebooks support short molecular-dynamics simulations directly in Colab, facilitating rapid structural validation and consistency checks before large-scale production runs.

## Data Availability

MartiniSurf is distributed as open-source software. The source code, documentation, and usage examples are publicly available at the MartiniSurf GitHub repository, https://github.com/BioKT/MartiniSurf, and at the project documentation Web site, https://biokt.github.io/MartiniSurf/. Input files, configuration examples, and workflow instructions required to reproduce the representative system-preparation protocols described in this work are available in the public repository and associated documentation.
